# Building First-Year Medical Students’ Skills in Finding, Evaluating, and Visualizing Health Information Through a “Debunking Medical Myths” Curricular Module

**DOI:** 10.1007/s40670-022-01541-w

**Published:** 2022-04-05

**Authors:** Katherine G. Akers, Ella Hu, Narmeen Rehman, Ho Jun Yun, Jacob Hoofman, Rachel Monconduit, Jennifer Mendez

**Affiliations:** 1 Shiffman Medical Library, Wayne State University, Detroit, MI USA; 2 School of Medicine, Wayne State University, Detroit, MI USA; 3 Internal Medicine, School of Medicine, Wayne State University, Detroit, MI USA

**Keywords:** Undergraduate medical education, Medical students, Evidence-based medicine, Public health, Health communication, Infographics

## Abstract

To provide an online service learning opportunity for medical students during the COVID-19 pandemic, medical faculty and librarians developed and implemented a “Debunking Medical Myths” module in which students learned to search for emerging medical literature, evaluate evidence, and use that evidence to create an infographics debunking a COVID-19-related myth for a non-medical audience. The resultant infographics are visually appealing and designed to make complex health information easy to understand. The module was well-received by students, who demonstrated a nuanced understanding of the use of infographics to convey health information, and students’ work was evaluated highly by community members.

**Supplementary Information:**

The online version contains supplementary material available at 10.1007/s40670-022-01541-w.

## Background

Running parallel to the devastating health outcomes of the COVID-19 pandemic is an “infodemic” of misinformation that undermines society’s ability to contain the virus and minimize its harm [1]. To help combat public misconceptions about COVID-19, physicians must share reputable health information with patients, friends and family, and community members [2]. However, the success of these efforts depends on the physicians’ ability to find and evaluate medical evidence [3] and communicate health information using language and formats that are easily understood by non-medical experts [4].

At Wayne State University (WSU)’s School of Medicine (SOM), medical students in their first (M1) and second (M2) years enroll in a Population, Patient, Physician, and Professionalism course that provides a holistic view of clinical practice through the lens of social, behavioral, and health systems sciences [5]. To aid the application of these concepts, students engage in 35 h of service learning per year, which typically involves in-person volunteering in free health clinics, schools, and other community organizations.

When the COVID-19 pandemic forced our medical curriculum to move mostly online, students’ usual service learning opportunities were temporarily unavailable. Thus, considering the need to combat COVID-19-related misconceptions among the public, the SOM service learning director collaborated with WSU medical librarians to develop and implement an online “Debunking Medical Myths” module in which students were taught to find and evaluate medical literature and use the strongest evidence to create infographics debunking COVID-19-related myths for a non-medical audience.

## Activity

The learning objectives of the “Debunking Medical Myths” module were to enable M1 students to (1) perform an effective literature search, (2) evaluate the strength of medical evidence, and (3) communicate complex medical information to a non-medical audience. The module consisted of asynchronous and synchronous content scaffolded across four steps throughout the Fall 2020 semester. Each step had an assignment completed by small groups of M1 students (50 groups with ~ 6 students each). Asynchronous content was delivered and assignments submitted via Canvas, and a synchronous session was held on Zoom. All module learning materials were reviewed and selected by librarian instructors based on their quality and perceived utility to successful module completion. Assignments were graded as “complete” or “incomplete” and accompanied by brief narrative feedback from a librarian instructor.

For Step 0, M1 students submitted a one-sentence statement of a medical myth related to COVID-19.

For Step 1, M1 students watched brief videos created by the National Center for Biotechnology Information and academic libraries on finding articles in PubMed, applying limits and filters, saving searches and setting up email alerts, and accessing full-text articles. The assignment was to perform a literature search in PubMed on the myth topic and to submit their final database search string.

For Step 2, M1 students read journal articles on evidence-based medicine and evaluating research quality and watched a YouTube video on the hierarchy of evidence. The assignment was to provide a ~ 250-word narrative reflection on how they evaluated the results of their literature search and at least three citations for peer-reviewed journal articles providing the strongest evidence against the myth.

For Step 3, M1 students visited websites with examples of health-related infographics, lists of digital tools for creating infographics, and information about writing in plain language. The assignment was to create an infographic in any static format (e.g., bookmark, pamphlet, poster) that debunked the myth for a non-medical audience.

To provide M2 students with an opportunity to practice mentoring skills, groups of M1 students were asked to send their assignments to corresponding groups of M2 students through dedicated channels in Microsoft Teams, and M2 students were asked to provide feedback.

Between step 1 and 2, M1 and M2 students attended a 1-hour synchronous session led by a librarian instructor, who provided an overview of the module; orchestrated three small-group break-out discussions about literature searching, evaluating evidence, and infographics design; and answered questions. A total of eight identical sessions were held to accommodate all students.

After module completion, groups of M1 students prepared a 10-min presentation consisting of two PowerPoint slides with recorded audio to describe the evidence speaking to the myth and present their infographics to an audience consisting of members of community organizations that provided service learning opportunities in previous years, older adults from WSU’s interprofessional team home visit program [6], subscribers to the DR-ED email listserv for medical educators, SOM students and family members, and SOM faculty and staff. To accommodate all groups, three 1-hour Zoom sessions were scheduled per day for 3 days, with each session containing ~ 6 presentations. Audience members were invited to evaluate each presentation on a 4-point Likert scale (4 = exceeds expectations, 3 = meets expectations, 2 = has room to grow, 1 = needs improvement).

Finally, a voluntary Qualtrics survey was administered to M1 students to ascertain their views on using infographics to communicate health information to a general audience and to obtain feedback on the module. Answers to close-ended questions were analyzed using descriptive statistics, and answers to open-ended questions were analyzed using informal thematic analysis.

## Results and Discussion

### Infographics

**Fig. 1:**
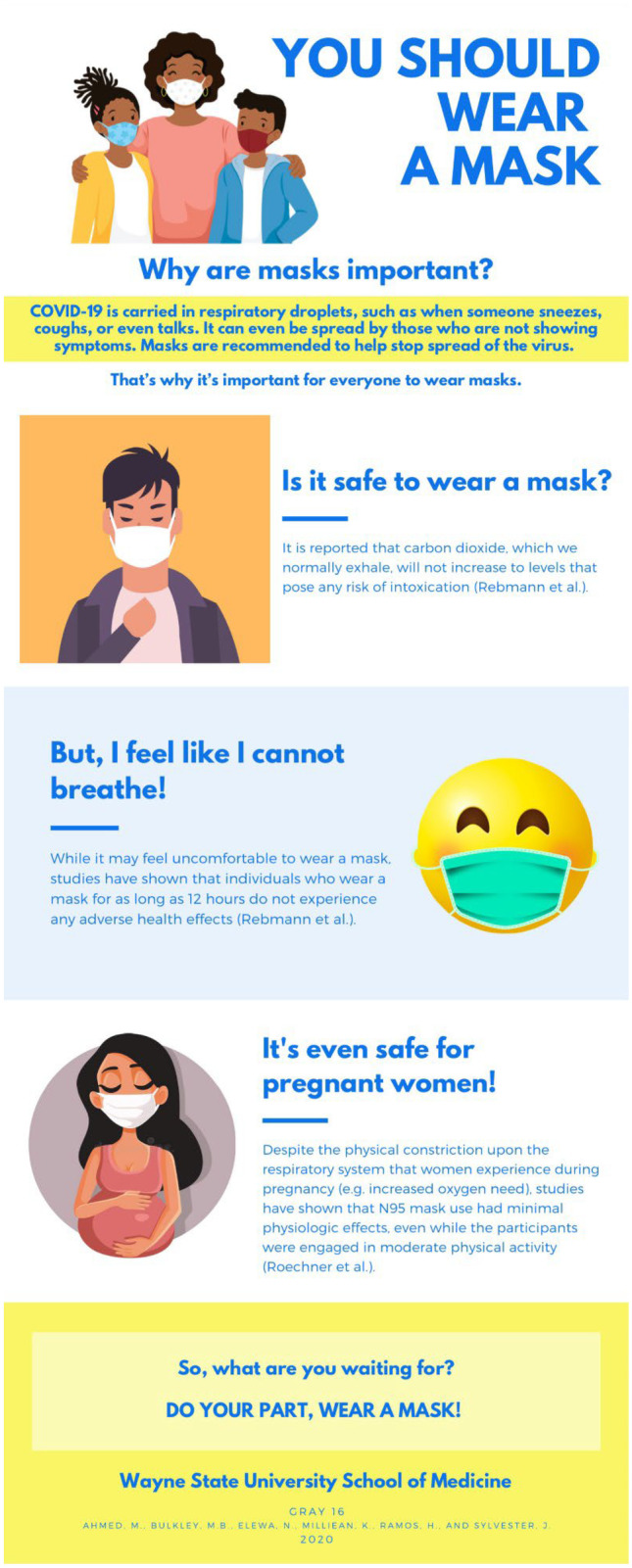
Example infographics created by medical students [7]

M1 students created visually appealing infographics debunking several COVID-19-related myths, such as the misconception that wearing a face mask is detrimental to one’s health (Fig. 1). After obtaining permission from students to share their infographics under a Creative Commons 0 license, which allows others to re-use the infographics without restriction, we published 44 infographics through WSU’s institutional repository (https://digitalcommons.wayne.edu/covidinfographics/). In the first 3 months after publication, the infographics were downloaded an average of 16.5 times each (range, 3 to 38 downloads). Thus, similar to initiatives at other universities [8–11], we utilized remote learning during the COVID-19 pandemic as an opportunity to equip medical students with the skills needed to synthesize medical evidence and create infographics combating the spread of COVID-19-related myths.

### Community Evaluation of Student Performance

Community members attending students’ presentations gave an average rating of 3.77 out of 4 (*n* = 919 total ratings), indicating that most presentations exceeded expectations.

### Students’ Views on the Use of Infographics

**Fig. 2:**
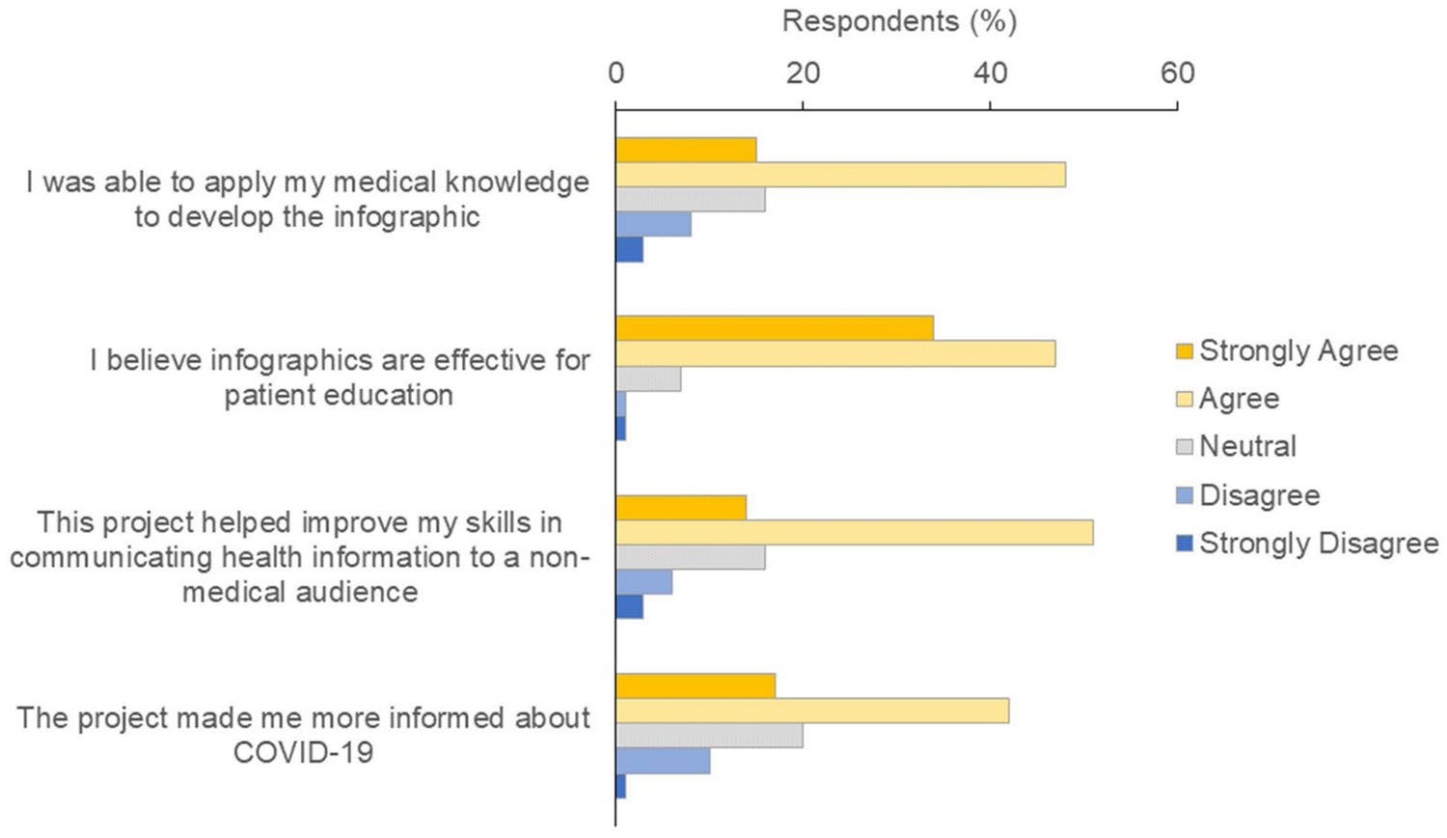
M1 students’ beliefs about the module and the use of infographics

Of the M1 students who completed the survey (*n* = 90), most agreed or strongly agreed that infographics are effective for patient education (Fig. 2). Students identified several “pros” of using infographics to convey health information to non-medical audiences, such as that infographics are easy to read and understand (42% of students), are concise and digestible (28%), attract attention (12%), can be disseminated to a wide audience (9%), and quickly convey “main points” (4%). However, students also identified several “cons” of infographics, such as that they contain a limited amount of information (26%), may over-simplify information (26%), may be misunderstood or misinterpreted (14%), are easy to disregard (8%), do not provide avenues for further research or learning (6%), and can be too complex or confusing (4%). To alleviate some of these concerns, students could receive more robust training in visual literacy [12] or be given more practical tips for designing infographics to increase the likelihood that they will be seen and understood by their intended audiences [13]. Also, graphic designers could be included as module instructors to provide more targeted instruction and assess students’ information visualization skills.

### Students’ Evaluation of Module

Most M1 students who completed the survey agreed or strongly agreed that they were able to apply their medical knowledge to develop the infographics, the module helped improve their communication of health information to a non-medical audience, and the project made them more informed about COVID-19 (Fig. 2).

**Fig. 3:**
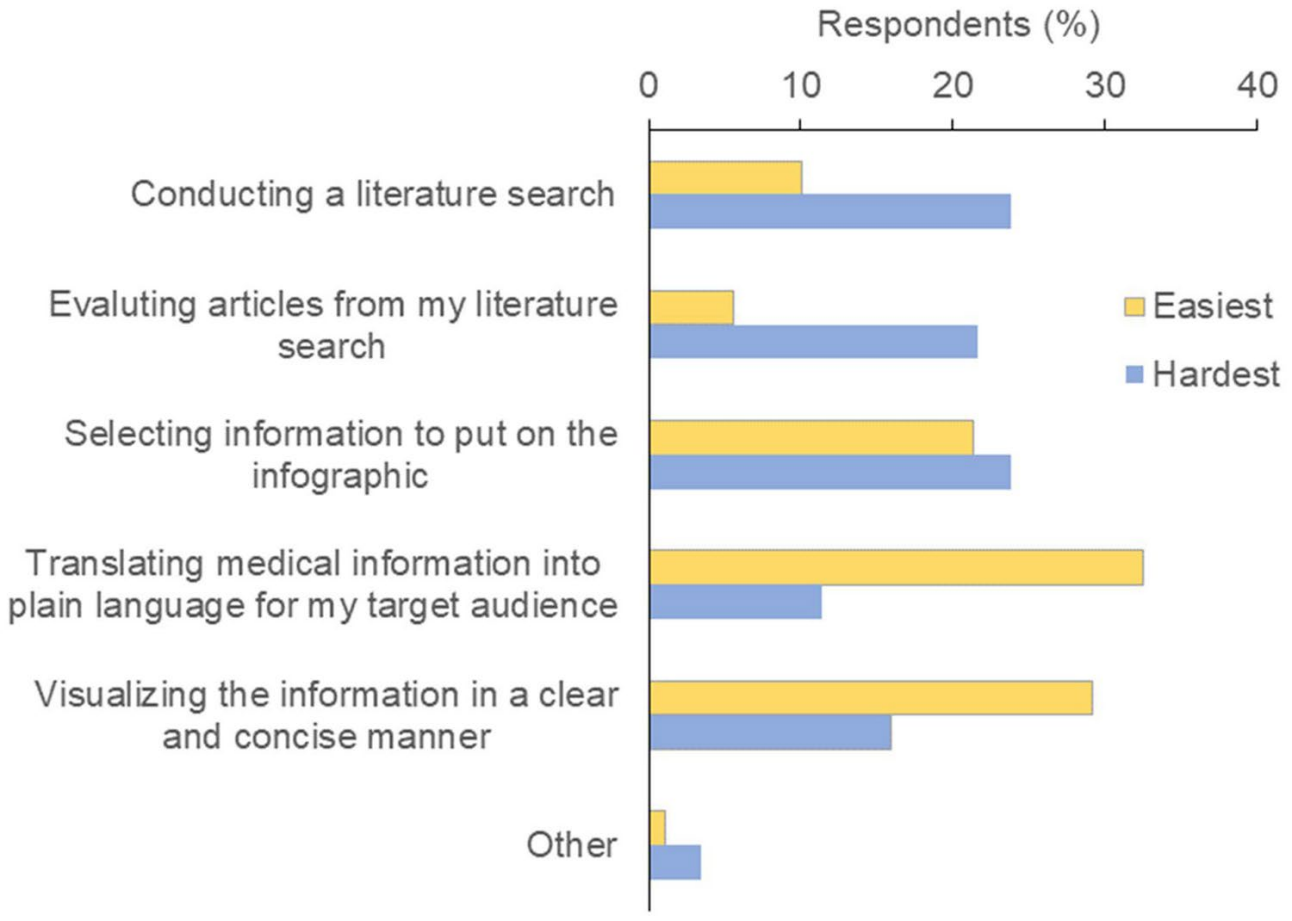
M1 students’ perceptions of the easiest and hardest aspects of the module

Students said the easiest aspects of the module were translating medical information into plain language and visualizing information in a clear and concise manner, whereas they found it more difficult to conduct a literature search, evaluate articles, and select information to include in the infographics (Fig. 3). Considering that medical students tend to overestimate their ability to search for and appraise evidence [14, 15], our finding that students deemed these aspects of the module most challenging suggests that future iterations of the module should incorporate more learning content and instructor support for these key components of evidence-based medicine.

Based on additional student feedback via the survey, we will further improve the module by shortening its duration from ~ 4 months to ~ 2 months and revisiting the module instructions to ensure their clarity. Also, because coordinating communication between M1 and M2 students was onerous and some M1 students reported that M2 students did not provide much feedback, we will not include M2 students in the next iteration but will consider ways of training M2 students to provide more constructive feedback in the future.

## Conclusion

Although WSU’s SOM is gradually returning to in-person learning in the wake of the COVID-19 pandemic, we are repeating the online “Debunking Medical Myths” module with M1 students in fall 2021 due to its positive reception by students and community members. Rather than debunking COVID-19-related myths, the next implementation of the module will focus on debunking myths about chronic illness, demonstrating the flexibility with which the module can be applied to different health topics. Furthermore, to enhance the potential for student-created infographics to positively impact public health, we aim to leverage WSU’s marketing and communication offices and our existing relationships with community organizations to more broadly disseminate the infographics in digital and physical formats so that they reach their intended audiences.

## Supplementary Information

Below is the link to the electronic supplementary material.

Supplementary file1 (DOCX 279 kb)

## Data Availability

Data are available in Online Resources: Online Resource 1: Module Content Online Resource 2: Student Created Resources (https://digitalcommons.wayne.edu/covidinfographics/)

## Code Availability

No code was employed.
