# Supplementary material for: Building First-Year Medical Students’ Skills in Finding, Evaluating, and Visualizing Health Information Through a “Debunking Medical Myths” Curricular Module
Source: Med Sci Educ. 2022 Apr 5;32(2):309–13. doi: 10.1007/s40670-022-01541-w (PMC8980765; doi:10.1007/s40670-022-01541-w)
Supplement: Supplementary file 1 — Supplementary file1 (DOCX 279 kb) [file 40670_2022_1541_MOESM1_ESM.docx]

**Online Resource 1: Module Content**

**Assignment Overview**

Multi-Step Assignment

Work together with the other members of your learning community group to create an infographic based on peer-reviewed articles that debunks a COVID-19-related medical myth for a patient/healthcare consumer audience.
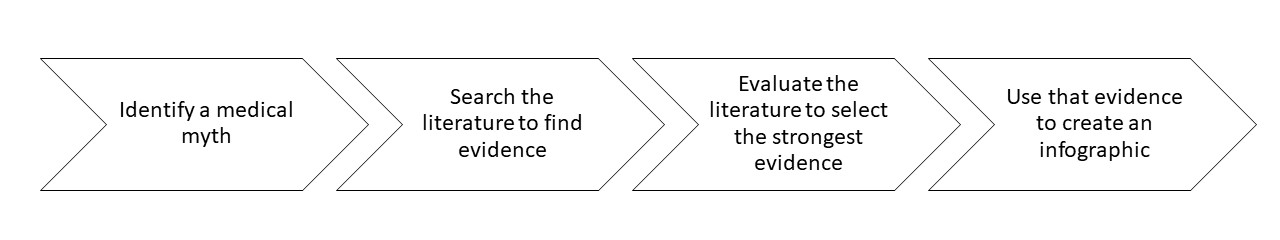


Step 0: Identify a medical myth related to COVID-19.

Step 1: Search the literature to find peer-reviewed articles that speak to the myth.

Step 2: Evaluate the literature to select the strongest evidence against the myth.

Step 3: Create a plain-language infographic that debunks the myth for a patient/healthcare consumer audience.

Your work for Step 2 and 3 will mentored and evaluated by M2 students. You will communicate with your corresponding M2 learning community group (i.e., M1 Blue 1 will communicate with M2 Blue 1) via a dedicated channel in Teams.

**Step 0: Identify a Medical Myth**

Together with the other members of your learning community group, identify a medical myth related to COVID-19. This medical myth will serve as the basis of your literature search (Step 1), literature evaluation (Step 2), and infographic creation (Step 3).

Examples of COVID-19-related medical myths include:

- <https://www.who.int/emergencies/diseases/novel-coronavirus-2019/advice-for-public/myth-busters>
- <https://www.mayoclinic.org/coronavirus-myths/art-20485720>
- <https://hartfordhealthcare.org/about-us/news-press/news-detail?articleId=26748&publicid=395>

You may choose whatever medical myth you want as long as it has a medical basis. However, when choosing your medical myth:

- Try to "think outside of the box" to select a unique medical myth that might not be addressed by the other learning communities.
- Consider a myth pertaining to one of the following areas of health: cardiovascular, chronic lower respiratory diseases, hypertension, obesity, HIV, diabetes, nutrition, or lead poisoning.
- Consider a medical myth situated in a particular area of health: [examples above]
- Start considering the ultimate target audience of your infographic (e.g., school-age children or adolescents, adults with chronic autoimmune disorders, retirement community residents).

**Step 0 Assignment: Identify a Medical Myth**

Group assignment

First, provide one sentence here identifying a medical myth related to COVID-19 that your learning community will debunk. Using text formatting, highlight the key concepts in the sentence that will be used as search terms in your literature search (Step 1).

Second, share your chosen medical myth with the M2 students in your corresponding learning community group i.e., M1 Blue 1 will communicate with M2 Blue 1) via your dedicated channel in Teams.

**Step 1: Search the Literature**

In this step, you will learn how to:

- conduct literature search on a topic in PubMed
- apply limits and filters to refine your search
- save your search and set up an email alert
- access full text articles

**Find Articles on a Topic**

Complete this short interactive tutorial on using PubMed to find articles on a topic.

<https://www.nlm.nih.gov/oet/ed/pubmed/quicktours/topic/index.html>

**Apply Limits and Filters in PubMed**

If your search retrieves too many results, you can apply limits or filters to narrow down the results. View this 1-minute video to learn how to apply limits and filters in PubMed.

[Searching in New PubMed: Applying Limits and Filters](https://youtu.be/QAlnJDXrvks)

Video credit: East Carolina University Laupus Health Sciences Library.

**Save Searches and Setup Email Alerts in PubMed**

Complete this short tutorial on how to save searches in PubMed to retrieve results in the future and set up email alerts to receive new publication alerts on your topic.

<https://www.nlm.nih.gov/oet/ed/pubmed/quicktours/alerts/index.html>

**Access Full Text Articles**

The article record page, which shows the article's abstract, links to full text access options in the upper right-hand corner. For example,


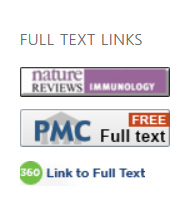


Sometimes a publisher makes the article open access to everyone, and sometimes the article is available in PubMed Central. Some articles only available through our library's subscription, which you'll be able to access by clicking "360 Link to Full Text". This video shows how to access full text articles through the "360 Link to Full Text".

[Connecting to Full Text Using Article Linker (360 Link to Full Text)](https://youtu.be/xr_lbjg1r6A)

**Step 1 Assignment: PubMed Search Strategy**

Group assignment:

Search PubMed to find peer-reviewed articles that speak to your medical myth.

- Connect to PubMed@WayneLinks to an external site. so you'll be able to access full text articles from journals subscribed by the library. To access full text articles, you will be asked to log in with your WSU AccessID and Password.
- Use search terms specific to your topic.
- Avoid using quotation marks and truncation (*).
- If your search retrieves too many results, apply filters in PubMed to narrow down the results.

Submit the search strategy for your final, refined PubMed search.

1. Click "Advanced" under the search box.
2. Scroll down to "History and Search Details".
3. Click the chevron icon ">" next to your final query in the "History and Search Details" to expand the Search Details.
4. Copy and paste the search strategy shown under "Query".

**Step 2: Evaluate the Literature**

Together with the other members of your learning community group, evaluate the results of your literature search and select the articles that provide the strongest evidence against the myth.

**Readings**

Rosenberg W, Donald A. Evidence based medicine: an approach to clinical problem-solving. BMJ. 1995;310(6987):1122-1126. doi:10.1136/bmj.310.6987.1122

Glasziou P, Vandenbroucke JP, Chalmers I. Assessing the quality of research. BMJ. 2004;328(7430):39-41. doi:10.1136/bmj.328.7430.39

**Video**

[The Hierarchy Of Evidence](https://youtu.be/0KP3ee4XHSA)

**Hierarchy of evidence**

**
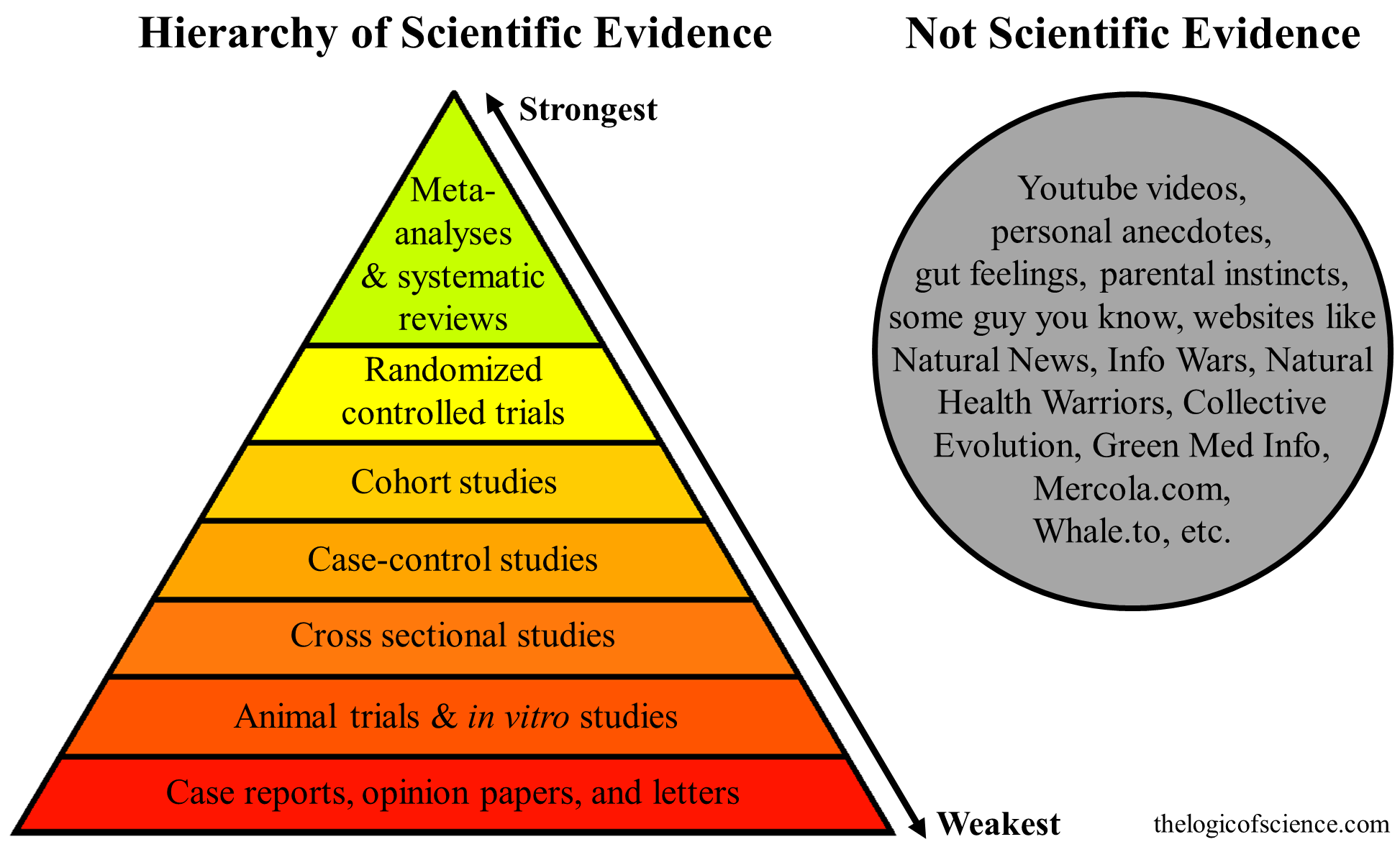
**

Image credit: <https://thelogicofscience.com/2016/01/12/the-hierarchy-of-evidence-is-the-studys-design-robust/>

**Step 2 Assignment: Literature Evaluation Reflection and References List**

Group assignment:

Provide a ~250-word narrative reflection on how you evaluated the results of your literature search and selected the articles that provided the strongest evidence against the myth. In the same file, also provide citations for at least three peer-reviewed articles, formatted in American Medical Association (AMA) (Links to an external site.) style, that provide evidence against the myth.

M2 students will help evaluate your performance on this assignment using the below rubric. You will communicate with your corresponding M2 learning community group (i.e., M1 Blue 1 will communicate with M2 Blue 1) via a dedicated channel in Teams (Team name: "Service Learning Medical Myth").

| **Needs Improvement** | **Has Room to Grow** | **Meets Expectations** | **Exceeds Expectations** |
| --- | --- | --- | --- |
| Evidence is unreliable and does not support health knowledge or practices. | Limited evidence to support health knowledge and practices. | General research supports broad health knowledge and practices. | Evidence-based research supports specific health knowledge and practices. |

**Step 3: Create an Infographic**

Together with the other members of your learning community group, create an infographic that debunks the medical myth for a patient/healthcare consumer audience. The infographic should be:

**Visually appealing**. Employ a simple, clean design to efficiently and effectively convey key information.

Examples of health-related infographics:

- [Google image search](https://www.google.com/search?rlz=1C1CHBF_enUS893US893&sxsrf=ALeKk02IS55f9-VgyXDrSI8U7xJvlw8KXw:1595427045236&source=univ&tbm=isch&q=health+infographic&sa=X&ved=2ahUKEwjC_67yhOHqAhWQQc0KHa4NBJkQsAR6BAgKEAE&cshid=1595427091840780&biw=1280&bih=566)
- [WHO examples](https://www.who.int/mediacentre/infographic/en/)
- [CDC examples](https://www.cdc.gov/socialmedia/tools/InfoGraphics.html)

Tools for creating infographics:

- [10 tools for creating infographics and visualizations](https://moz.com/blog/10-tools-for-creating-infographics-visualizations)
- [12 tools to create an infographic in 30 minutes (design skills or not)](https://buffer.com/library/infographic-makers/)

**Easily understood**. Use plain language. Replace medical jargon with language that is clearer and more meaningful to a lay audience.

Resources:

- [“Medspeak” can shut down effective communication with patients](https://www.ama-assn.org/delivering-care/patient-support-advocacy/medspeak-can-shut-down-effective-communication-patients)
- [What did my doctor say?](https://www.mlanet.org/page/what-did-my-doctor-say)
- [CDC everyday words for public health communication](https://www.cdc.gov/other/pdf/everydaywords-060216-final.pdf)
- [University of Michigan Library's Plain Language Medical Dictionary](https://apps.lib.umich.edu/medical-dictionary/)

**Designed in any static format**, such as a one-page flyer, pamphlet, postcard, bookmark, or wall poster.

**Step 3 Assignment: Infographic**

Group assignment:

Submit a pdf version of an infographic that debunks the myth in plain language for a patient/healthcare consumer audience. This infographic can be designed in any format (e.g., one-page flyer, pamphlet, postcard, bookmark, wall poster).

M2 students will help evaluate your performance on this assignment based on the following questions: How effectively and efficiently does the infographic convey information in plain language that debunks the medical myth for a patient/healthcare consumer audience? What are the strengths and weaknesses of the infographic? You will communicate with your corresponding M2 learning community group (i.e., M1 Blue 1 will communicate with M2 Blue 1) via a dedicated channel in Teams (Team name: "Service Learning Medical Myth").

**Online Resource 2: Student Created Resources**

**https://digitalcommons.wayne.edu/covidinfographics/**
